# Motivation of Community Health Workers in Diagnosing, Treating, and Referring Sick Young Children in a Multicountry Study

**DOI:** 10.1093/cid/ciw625

**Published:** 2016-12-06

**Authors:** Armande K. Sanou, Ayodele S. Jegede, Jesca Nsungwa-Sabiiti, Mohamadou Siribié, IkeOluwapo O. Ajayi, Asaf Turinde, Frederick O. Oshiname, Luc Sermé, Vanessa Kabarungi, Catherine O. Falade, Josephine Kyaligonza, Chinenye Afonne, Andrew Balyeku, Joëlle Castellani, Melba Gomes

**Affiliations:** 1 Groupe de Recherche Action en Santé, Ouagadougou, Burkina Faso; 2 Department of Sociology, Faculty of the Social Sciences; 3 Department of Epidemiology and Medical Statistics; 4 Department of Health Promotion and Education, Faculty of Public Health; 5 Department of Pharmacology and Therapeutics; 6 Epidemiology and Biostatistics Research Unit, Institute of Advanced Medical Research and Training, College of Medicine, University of Ibadan, Nigeria; 7 Child Health Division, Ministry of Health, Kampala, Uganda; 8 Department of Health Services Research, School for Public Health and Primary Care, Maastricht University, The Netherlands; 9 UNICEF/UNDP/World Bank/WHO/Special Programme for Research & Training in Tropical Diseases, World Health Organization, Geneva, Switzerland

**Keywords:** community health worker, motivation, retention, volunteers, Africa

## Abstract

***Background.*** Community health workers (CHWs) are an important element of care provision for a wide range of conditions, but their turnover rate is high. Many studies have been conducted on health workers’ motivation, focusing on formal sector staff but not CHWs. Although CHWs are easy to recruit, motivating and retaining them for service delivery is difficult. This article investigates factors influencing CHW motivation and retention in health service delivery.

***Methods.*** Quantitative and qualitative data were collected to identify the key factors favoring motivation and retention of CHWs as well as those deterring them. We interviewed 47, 25, and 134 CHWs in Burkina Faso, Nigeria, and Uganda, respectively, using a structured questionnaire. Focus group discussions (FGDs) were also conducted with CHWs, community participants, and facility health workers.

***Results.*** Except for Burkina Faso, most CHWs were female. Average age was between 38 and 41 years, and most came from agricultural communities. The majority (52%–80%) judged they had a high to very high level of satisfaction, but most CHWs (approximately 75%) in Burkina Faso and Uganda indicated that they would be prepared to leave the job, citing income as a major reason. Community recognition and opportunities for training and supervision were major incentives in all countries, but the volume of unremunerated work, at a time when both malaria-positive cases and farming needs were at their peak, was challenging.

***Conclusions.*** Most CHWs understood the volunteer nature of their position but desired community recognition and modest financial remuneration.

***Clinical Trials Registration.*** ISRCTN13858170.

It has long been known that village or community health workers (CHWs) can play a major role in reducing malaria morbidity and mortality in areas where access to skilled care and commodities is poor [1–3]. Community health workers are the backbone of health coverage providing health services to the healthcare pyramid at its base; they therefore serve the greatest number of people in each country and perform multiple health tasks, from case management to environmental sanitation far from healthcare facilities. Although trained CHWs do not replace the need for highly skilled healthcare workers, they bridge the gap between the formal health system and the community by screening patients who can safely be treated in the community and referring those who should obtain specialized care. They are provided with medical supplies and supervision after initial training, which ranges from several hours to several days or months. However, supplies and supervision can be inconsistent and erratic, there is no career structure, and in sub-Saharan Africa they are invariably unpaid and have an ambiguous role and the lowest status within the health system [4–7]. Consequently, CHWs have a high attrition rate [8]. Nevertheless, major improvements in health have been achieved because of CHW involvement [3].

Our study using CHWs to diagnose and treat malaria documented high levels of coverage achieved by CHWs in their communities after training and without excessive expenditure of CHW time [9]. CHWs were chosen by their communities, were trained with job aids, and were supervised by the health system and the research team as though they were paid as regular employees. The sole exception, in keeping with country policy, was in Burkina Faso, where CHWs were permitted to charge a modest fee to each treated patient.

The voluntary nature of the CHW program and the apparent success of the work in all countries led us to explore the motivation of CHWs in the service of their community. The objective was to understand why CHWs were inspired to work for their communities. Unlike trained health workers working in the formal health sector, who are motivated through financial means and who have career development pathways, health infrastructure support, and greater likelihood for personal recognition [10, 11], it was not clear what the motivation was for Agents de Santé Communautaire in Burkina Faso, community medicine distributors in Nigeria, or village health teams in Uganda (hereinafter called CHWs).

## METHODS

### Study Settings and Population

The study was implemented in Burkina Faso, Nigeria, and Uganda and was carried out within the last 4 months of the intervention study [10].

### Data Collection Methods

We employed a mixed-methods approach using a combination of qualitative and quantitative techniques. In Burkina Faso, we interviewed all 47 CHWs in a structured survey. In addition, 2 focus group discussions (FGDs) were held with a group of male and female CHWs (7 men and 5 women). Three communities were randomly selected, and in each, 2 additional FGDs were carried out that included caregivers and community leaders, 1 for each gender. One more FGD was conducted with all health workers involved in the intervention, including facility staff. In Nigeria, the same procedure was followed for Badeku and Akanran axes, with 25 CHWs involved in the structured survey.

In Uganda, a slightly different approach was used. Each village normally has 2 CHWs, and we selected 1 or both for interview if they were available (*n* = 134). In addition, 24 CHWs were randomly selected and grouped into 4 FGDs of 6 CHWs participants per group. Each discussion session also included individuals from different communities to increase representation of the communities involved.

#### Conduct of the FGDs

FGDs were conducted by trained research assistants who were supervised by social scientists in each country. Research assistants were trained for 2–3 days prior to data collection. The structured surveys were piloted before use.

### Data Analysis

The quantitative data were analyzed using SPSS software version 16.0. Transcribed FGDs were categorized and analyzed according to study themes at each country level by the research teams. The qualitative narratives were reviewed by anthropologists without specialized software in Burkina Faso and Uganda and using Nvivo 8 software in Nigeria.

A single person in each country coded the transcripts. All interviewees were assigned numbers, so that their role, location, and associated health facility were anonymized. The findings were integrated and triangulated on presentation.

### Ethical Approval

Ethical approval for the study was granted by the World Health Organization Ethics Review Committee and by the appropriate national and institutional ethical review boards of each participating country. Informed consent was obtained from the CHWs, community members and leaders, and the caregivers who participated in the study.

## RESULTS

### Quantitative Results

All CHWs were female in Nigeria and 73% were female in Uganda, compared with 21% in Burkina Faso (Table 1). The mean age was similar: 38 years in Burkina Faso, 41 years in Uganda, and 42 years in Nigeria, and the majority of CHWs were married. Between 32% and 55% of CHWs indicated that they were not originally from the area. Every CHW was literate, and education attained was highest in Uganda.

**Table 1.** Characteristics of Community Health Workers Interviewed

Characteristic	Burkina Faso (n = 47)	Nigeria (n = 25)	Uganda (n = 134)
Age			
Mean, y (range)	38 (18–66)	42 (25–64)	41 (25–64)
<30 y	13 (27.7)	1 (4.0)	6 (4.5)
30–45 y	18 (38.3)	17 (68.0)	84 (62.7)
>45 y	16 (34.0)	7 (28.0)	40 (29.9)
Missing	…	…	4 (3.0)
Gender			
Male	37 (78.7)	…	36 (26.9)
Female	10 (21.3)	25 (100.0)	98 (73.1)
Education level			
Primary	24 (51.0)	16 (64.0)	45 (33.6)
Secondary	21 (44.7)	9 (36.0)	84 (62.7)
Tertiary	…	…	2 (1.5)
Other	2 (4.3)	…	…
Missing	…	…	3 (2.2)
Marital status			
Single	6 (12.8)	…	2 (1.5)
Married	40 (85.1)	20 (80.0)	120 (89.6)
Divorced/separated	…	2 (8.0)	4 (3.0)
Widow	1 (2.1)	3 (12.0)	7 (5.2)
Missing	…	…	1 (0.7)
Originating from area			
No	32 (68.1)	15 (60.0)	40 (29.9)
Yes	15 (31.9)	8 (32.0)	74 (55.2)
Missing	…	2 (8.0)	20 (14.9)
Principal source of income			
Agriculture/farming/husbandry	37 (78.8)	11 (44.0)	117 (87.3)
Paid worker	1 (2.1)	…	2 (1.5)
Other business/shop/tailor	5 (10.6)	12 (48.0)	15 (11.2)
Other	4 (8.5)	2 (8.0)	…
Years of experience as CHW			
<1 y	13 (27.7)	1 (4.0)	8 (6.0)
1 to <2 y	…	5 (20.0)	15 (11.2)
2 to <3 y	7 (14.9)	7 (28.0)	33 (24.6)
>3 y	27 (57.4)	12 (48.0)	78 (58.2)
Hours CHW reported working per week			
<5 h	1 (2.1)	8 (32.0)	23 (17.2)
5–10 h	30 (63.8)	14 (56.0)	43 (32.1)
11–15 h	16 (34.0)	3 (12.0)	51 (38.0)
Other paid work	…	…	10 (7.5)
Missing	…	…	7 (5.2)

Data are presented as No. (%) unless otherwise indicated.

Abbreviation: CHW, community health worker.

Table 1 shows that agriculture and farming was an important source of CHW family income, varying from 44% in Nigeria to 87.3% in Uganda. Within the majority of locations, CHWs had >3 years’ experience: 57.4% in Burkina Faso, 58% in Uganda, and 48% in Nigeria. Overall, 42.2% of CHWs stated that they devoted 5–10 hours per week to provide healthcare to children, although the volume varied by country: 63.8% in Burkina Faso, 56% in Nigeria, and 32% in Uganda.

The reasons most often cited as influencing the decision to accept CHW work were training opportunities (>70% in all countries) and the opportunity to serve their communities (>74%). Social knowledge and understanding, and improved status in the community, were cited in Uganda; supplementary income and supervision were cited in Burkina Faso.

### Qualitative Results

### Perceptions and Opinions About Volunteering Time and CHWs’ Motivation

#### Community Perception

Informants interviewed in each of the 3 countries were surprised to learn that CHWs volunteered. When informed, community members considered CHWs as unwise for having left their livelihood to undertake an activity that brought no income. However, they recognized and commended CHWs for their contribution and were unanimous that CHWs need support in their efforts for the community. One caregiver in Burkina Faso said:

They are sought; we disturb them always. They arrive in the fields; barely bent, and we recall them for sick children. They return. We wake up them in the night. One really has to appreciate their sacrifice, as if they were full-time nurses. Knowing they are not, and that they are dedicated, it is normal to appreciate them. This will give them benefit and improve their performance.

Most community members considered that appreciation/financial reward should come from the government or nongovernmental organizations.

#### Health Staff Perception

Facility staff valued the work of the volunteers. They had apparently undertaken unsuccessful steps to obtain community support for CHWs through food donations at harvest or during the year, but many communities did not believe that CHWs volunteer. One health worker (HW) in Matsyoro Uganda perceived voluntarism as beneficial and uplifting. She said it gives “*a sense of accomplishment …. While no monetary compensation is received, many will tell you that their work and experiences gained as a volunteer were worth way more than any money  …  Volunteer work makes them feel good. It builds self-confidence and lifts up the spirits*.” But other HWs recognized the need to pay CHWs because volunteering took time away from their main source of income.

HWs also emphasized the importance of financial support and certificates to further enhance their work. “*A congratulatory letter, a card, it strengthens [motivation]. The fact that they hang [the card]  …  gives value to the person.*”

### CHWs Perceptions and Opinions About Incentives

In Burkina Faso, the CHWs were allowed to charge 30 West Africa CFA francs (XOF) US Dollars (USD) $.05 or 50 XOF (USD $.08) per treatment, by age and obtained cash (5000 XOF [USD $10]) for transport every month. The CHWs from Uganda received nonmonetary incentives (T-shirts, bicycles, torches, batteries, and a small transport reimbursement of 5000–10 000 Uganda Shillings [UGX] [USD $2–USD $5] for quarterly meetings at the health facility). In Nigeria, the CHWs were given food during festive periods, and USD $10 transport reimbursement for each meeting.

When asked about their motivation as CHWs, 53.2% in Burkina Faso thought that they were well motivated, compared with 52% in Nigeria and 33.6% in Uganda. In regard to their general level of satisfaction as a CHW, the majority of CHWs declared that it is high in Burkina Faso (57.4%) and Nigeria (48%), and moderate (44%) in Uganda. The CHWs perceived that their work serves the community and contributes to its development. According to a CHW in Nigeria: “*People think that we get paid for our service. Truly, we whole-heartedly take it as job for the love of our community  …  It is just a humanitarian service*.”

### Factors/Source of the CHWs’ Motivation

In our questionnaire on motivation, relatively few CHWs indicated that they were unhappy working as a CHW (range, 0%–27.7%) (Table 2), and their responses to a question on general level of satisfaction when working as a CHW tended to correspond with this pattern: high or very high satisfaction was >80% in Burkina Faso and Nigeria and 52.9% in Uganda. In Burkina Faso and Uganda, nearly three-quarters of CHWs said they had received financial benefits (mainly cash and work materials) in response to a multiple-choice question on the type of benefits received, but only 12% claimed any benefits in Nigeria.

**Table 2.** Community Health Workers’ Level of Satisfaction in Their Work

Category/Question	Burkina Faso (n = 47)	Nigeria (n = 25)	Uganda (n = 134)
Description of CHW motivation			
Very high	4 (8.5)	13 (52.0)	45 (33.6)
High	5 (10.6)	7 (28.0)	25 (18.7)
Good	25 (53.2)	5 (20.0)	37 (27.6)
Unhappy	13 (27.7)	…	27 (20.1)
General level of satisfaction as a CHW			
Very high	27 (57.5)	12 (48.0)	27 (20.1)
High	16 (34.0)	10 (40.0)	44 (32.9)
Medium	4 (8.5)	3 (12.0)	33 (24.6)
Low	…	…	29 (21.6)
Missing	…	…	1 (0.8)
CHW response whether they received material benefits			
No	12 (25.5)	22 (88.0)	31 (23.1)
Yes	35 (74.5)	3 (12.0)	103 (76.9)
Material benefits obtained^a^			
Money	35 (74.5)	2 (8.0)	42 (31.3)
Food	…	1 (4.0)	2 (1.5)
Bicycle	…	…	55 (41.0)
Work material	8 (17.0)	…	…
T-shirts/badge/vest	4 (8.5)	3 (12.0)	5 (3.7)
Factors motivating decision to accept CHW work^a^			
Supplementary income	30 (63.8)	3 (12.0)	33 (24.6)
Good working conditions	23 (48.9)	1 (4.0)	55 (41.0)
Training opportunities	41 (87.2)	18 (72.0)	111 (82.8)
Status in the community	21 (44.7)	3 (12.0)	71 (53.0)
Opportunity to serve the community	35 (74.5)	21 (84.0)	107 (79.9)
Social knowledge/understanding	23 (48.9)	3 (12.0)	86 (64.2)
Supervision	27 (57.4)	1 (4.0)	55 (41.0)
Community support (food, work in the fields)	1 (2.1)	…	13 (9.7)
To improve health of children	…	1 (4.0)	…
Material benefits	…	…	1 (0.7)

Data are presented as No. (%).

Abbreviation: CHW, community health worker.

^a^ Multiple-choice question.

During the FGDs, many CHWs cited child health as one of the most important motivating factors. They accepted the job because of their contribution to the reduction of severe malaria and death, and the opportunity of making medicines available to children close to the home, at no cost or at a low cost.

*Before, we ignored  …  malaria  …  We know now what malaria is, what its consequences are in the children. In the past we ignored all about the rapid diagnosis test and now we know how to do it. That contributed to our skills, and improved our children's health.* — Female CHW, Burkina Faso

Most CHWs mentioned that supervision gives them an opportunity to refresh skills and boosts esteem and morale.

### Factors Affecting Retention of CHWs

Three-quarters of CHWs in Burkina Faso and Uganda indicated that they would be prepared to leave the job as CHW; income and opportunities for training were considered to be major incentives in both countries, and the amount of work was considered to be a major challenge (Table 3). Most (88%) CHWs in Nigeria indicated that they would not consider leaving their position as a CHW but cited opportunities for training and serving the community as major incentives desired. Among those who had thought about leaving, dissatisfaction related to compensation was the main reason.

**Table 3.** Difficulties and Challenges Faced by Community Health Workers

Category/Question	Burkina Faso (n = 47)	Nigeria (n = 25)	Uganda (n = 134)
Do you imagine abandoning this job?			
No	12 (25.5)	22 (88.0)	31 (23.1)
Yes	35 (74.5)	3 (12.0)	103 (76.9)
Incentives desired by CHW^a^			
Supplementary income	46 (97.9)	3 (12.0)	125 (93.3)
Good terms of working conditions	34 (72.3)	1 (4.0)	97 (72.4)
Opportunities of training	40 (85.1)	18 (72.0)	111 (82.8)
Position in the community	7 (14.9)	3 (12.0)	86 (64.2)
Opportunity of serving the community	17 (36.2)	21 (84.0)	85 (63.4)
Social knowledge	16 (34.0)	3 (12.0)	85 (63.4)
Supervision	30 (63.8)	1 (4.0)	91 (67.9)
Community help (food, work in the field)	11 (23.4)	…	45 (33.6)
Difficulties/challenges faced by CHW^a^			
Amount of work	41 (87.2)	6 (24.0)	134 (100.0)
Absence of motivation	10 (21.3)	2 (8.0)	119 (88.8)
Absence of help/support in the community	3 (6.4)	6 (24.0)	77 (57.5)
Problems of stockouts of drugs	3 (6.4)	…	47 (35.1)
Having to go at night to a patient (in areas without electricity)	1 (2.1)	1 (4.0)	…
Distance to visit child (bicycles are worn out)	…	…	5 (3.7)
Poor health or physical condition	…	1 (4.0)	…
Not enough patients	…	4 (16.0)	16 (11.9)
Marriage, divorce, new work offer, or opportunities in another community	…	5 (20.0)	20 (14.9)

Data are presented as No. (%).

Abbreviation: CHW, community health worker.

^a^ Multiple-choice question.

All CHWs interviewed agreed that lack of income was a challenge. The small amount of transport refunds given for replenishment of supplies, frequent medicine stockouts, the volume of work, receiving patients at night with no electricity, lack of community support, and community refusal to appreciate that CHWs were volunteers were seen as disincentives.

### Factors Influencing Loyalty/Attrition

Treatment efficacy and access to medicines and blood test kits for case management were frequently mentioned as major incentives.

The prestige and social responsibility attached to the service as CHWs and pride in being called “nurse or doctor” also influenced loyalty and commitment:

*The work of volunteers is cherished and we…are very valued  …  the way they bring their sick children to us for care is amazing  …  it was not that we attended a medical or nursing school that made them bring their sick children  …  They bring their sick children, they get well and so they call us  …  nurse, and that is another form of respect.* — FGD with CHWs, Nigeria

Many CHWs benefit from family understanding and support. Families encourage CHWs to care for children even if they are working in the fields or they are woken up at night with someone knocking on their door. Health workers independently confirmed the importance of family support and commented that a family member would come in search of the CHW because a patient was waiting.

### Barriers and Challenges

The quantitative survey cited volume of work as a main difficulty: 87% in Burkina Faso, 24% in Nigeria, and 100% in Uganda mentioned this constraint and the lack of remuneration (Table 3).

FGDs confirmed several difficulties faced by CHWs. The fact that CHWs are volunteers implies that the CHWs have other work and source of income, yet the CHWs have to be available when a patient is ill.

*The farming season  …  coincides with the period when malaria is important  …  It happens that you treat two to three patients before returning to your field.  …  In view of the child's health status you do not even hesitate. Then the next day you realize that you have delayed in your field. So, you are going to employ laborers to work  …  Certainly you helped the community, but how to solve the delay in your field work?  …  The rainy season  …  is the intense period of our activities.* — Male CHW, FGD, Djalakoro, Burkina Faso

Other constraints mentioned were refusal of caregivers to comply with referral advice or failure to complete medication, or to accept that the child should not be treated when the rapid diagnostic test (RDT) is negative.

Migration for other paid employment, educational opportunities, and filial responsibilities or sickness were mentioned as a barrier to the program. In Uganda, failure to visit homes to check on their clients in the villages because of a lack of or damaged bicycles, or stockouts of commodities (cited by 35%), was a disincentive that affected CHW morale and their relationship with clients. They regretted the short supervision time of health workers to discuss their difficulties.

### CHW Expectations Regarding Income

In FGDs, all the CHWs in each country agreed that a salary would motivate them to work harder with honesty. CHWs wanted to have supplementary incomes as well as training opportunities: 85%–98% in Burkina Faso, 72%–80% in Nigeria, and 83%–93% in Uganda mentioned this in FGDs. Various terms of payment were proposed, a suggestion of USD $40–USD $60 per month in Burkina Faso and USD $10–USD $12 in Uganda. The majority of CHWs reported (72% in Burkina Faso and Uganda) that some materials would help their performance (good bicycles, lamps, bags, rubber boots, umbrellas) or reinforce community respect and credibility (T-shirts or bibs, work cards, certificates).

## DISCUSSION

As long as CHWs remain voluntary, their effectiveness in community-based work depends upon their motivation and retention. High attrition reduces continuity between the CHW and community and increases recurrent costs of selecting and retraining new CHWs. Experience acquired is lost. In the wake of a successful program to increase access to diagnosis and treatment for malaria through CHWs in 3 countries (Burkina Faso, Nigeria, and Uganda), this study explored the motivations, challenges, and expectations of CHWs within different health contexts. Overall, the study showed that although existing CHWs had high motivation, the majority in Uganda and Burkina Faso, which had the most patients, said that they could imagine leaving the program.

Status and recognition in the community in which they work, receipt of individual training and supervision, and commitment to the well-being of children were identified as factors positively influencing CHWs, confirming that context affects their motivation and retention [12–15]. Monetary and nonmonetary incentives were perceived to be critical. CHWs derived satisfaction from community recognition or appreciation and took pride in being called a nurse and felt needed when there was high demand for their services [5]. Equally, they were demoralized when parents or community members did not appreciate that their services were unpaid, and that they were providing social services at some personal cost.

CHWs understood that their role was voluntary, but they wished for material benefits, community support, and supplies to be commensurate with their roles. Very small items increased their sense of pride and self-worth: a certificate of training, an identification badge, a T-shirt, working bicycles, and the ability to talk to their supervisors and be appreciated. Appropriate and regular supplies strengthened their role and interactions with guardians. Despite awareness about the unpaid nature of the job, they would often lag on their farm duties. When they were not paid for their time, they had a conflict with time needed for farming and the time needed for child care, especially because the rainy season increased malaria and farm chores simultaneously. Some CHWs hoped their financial situation might change in the future because of current sacrifices.

Other challenges were links to the community and links to the health service. They mentioned lack of cooperation of guardians (poor adherence with the correct dose, compliance with referral advice, and understanding of RDT-negative results), inadequate clarity of their role and links with formal health services, commodity stockouts, lack of and short supervisory time, limited refresher training (notably in Uganda), and inadequate transport compensation for replenishment of supplies or attending supervisory meetings.

In this study, CHWs were trained but not paid, except in Burkina Faso where guardians of sick children contributed toward the cost of treatment as part of each transaction. CHWs provided voluntary services to underserved populations but were evaluated and held accountable as if they were salaried workers and were aggrieved to learn that community members did not understand or appreciate the volunteer nature of their contribution. Although the time they spent on healthcare was small [16], the cost savings to the community were significant [9] in a context where formal health services were limited. The existing evidence overwhelmingly suggests that CHWs are a good investment in providing access to and coverage of basic health services.

The incentives that most affected CHWs were community recognition and status, regular training, and provision of supplies. These are reasonable and mutually beneficial requests to improve motivation and reduce attrition. Strengthening the health system to include CHWs is where the greatest gains in health can be made to the greatest number of people. CHWs are no richer than other community members and need financial incentives, career development paths, and support for the work they do. Financial incentives are not the only response, but lack of remuneration for their time and effort, either directly from the communities they serve or from Ministries of Health, is no longer justifiable.

There were limitations to this study. It is possible that the responses reflect what participants thought they should say instead of what they believe. Data collection coincided with the harvest season in Burkina Faso and with the presidential election in Uganda, and this caused individual interviews and FGDs to be conducted at the end of the day; therefore, there were many interview deferrals, which may have had an impact on responses. In Nigeria, the views of facility staff could not be obtained.
